# Joint health status in people with moderate hemophilia A: a cross-sectional multicenter study

**DOI:** 10.1016/j.rpth.2025.102737

**Published:** 2025-03-19

**Authors:** Ilenia Lorenza Calcaterra, Federico Picasso, Federica Valeri, Erminia Baldacci, Mariasanta Napolitano, Cornelia Guerrino, Ezio Zanon, Cristina Santoro, Sergio Siragusa, Carlo Martinoli, Matteo Nicola Dario Di Minno

**Affiliations:** 1 Department of Clinical Medicine and Surgery, Regional Reference Centre for Coagulation Disorders, Federico II University, Naples, Italy; 2 Department of Health Sciences, Università di Genova, Genova, Italy; 3 Regional Centre for Hemorrhagic and Thrombotic Diseases, Azienda Ospedaliera UNiversitaria Città della Salute e della Scienza, Turin, Italy; 4 Haematology, Department of Translational and Precision Medicine, “Sapienza” University, Rome, Italy; 5 Department of Health Promotion, Mother and Child Care, Internal Medicine and Medical Specialties, University of Palermo, Palermo, Italy; 6 Haematology Unit and Rare Disorders, Hospital “V.Cervello,” Palermo, Italy; 7 Haemophilia Centre, General Medicine, Padua University Hospital, Padua, Italy

**Keywords:** arthropathy, joint score, moderate hemophilia, prophylaxis, ultrasound

## Abstract

**Background:**

The prevalence of arthropathy in people with moderate hemophilia A (mHA) is highly variable. People with mHA are often undertreated, and this may lead to joint damage and worsen their quality of life.

**Objectives:**

The aim of the present study was to evaluate joint status in mHA by means of point-of-care ultrasound (PoCUS) and clinical examination.

**Methods:**

Consecutive people with mHA receiving on-demand replacement treatment underwent a clinical examination of joint status according to the Hemophilia Joint Health Score (HJHS) protocol. On the same day, all patients underwent a PoCUS assessment according to the Hemophilia Early Detection by UltraSound (HEAD-US) protocol.

**Results:**

A total of 51 subjects were included. The median HJHS score was 2.0 (IQR, 0-3.0). A 0 to 1 HJHS score was found in 23 people with mHA (45.1%), between 2 and 3 in 17 (33.3%) and >3 in 11 (21.6%). The median HEAD-US score was 2.0 (IQR, 1-7), and a statistically significant correlation between HJHS and HEAD-US was found (rho = 0.732; *P* < .001). Osteochondral damage was found in 21.6% of patients, and hypertrophic synovium (HS) was found in 29.4%. Among those reporting a 0 to 1 HJHS score, 13.0% showed HS. Analysis at the joint level showed that the most commonly affected joint was the ankle, both for osteochondral damage and the presence of HS.

**Conclusion:**

Our study suggests that the prevalence of arthropathy changes in people with mHA receiving on-demand treatment is not negligible and that PoCUS is able to detect osteochondral damage as well as HS in this clinical setting. A more extensive screening of the joint status could be useful to tailor treatment and improve outcomes in mHA.

## Essentials

• Arthropathy in people with moderate hemophilia A is often underdiagnosed and undertreated.
• We evaluated the prevalence of arthropathy in 51 people with moderate hemophilia A.
• We found a significant prevalence of arthropathy in people with moderate hemophilia treated on-demand.
• We highlighted that PoCUS is able to detect signs of disease activity and osteochondral damage.

## 1 Introduction

Hemarthrosis represents the most common type of bleeding in subjects with hemophilia A (HA) [[1], [2], [3]] and typically involves large synovial joints such as knees, elbows, and ankles [1,2]. Recurrent hemarthrosis triggers chronic arthropathy as result of a concomitant inflammatory and degenerative process [2,4]. Hemophilic arthropathy is a common major complication leading to disability, chronic pain, and impaired quality of life. Based on both clinical (the Gilbert Orthopedic Joint Score) [5,6] and radiologic (Patterson Score) [7] assessments, up to 85% of people with severe HA (factor [F]VIII levels <1%) develop hemophilic arthropathy, mainly in the absence of adequate prophylaxis [8]. People with moderate HA (mHA; FVIII levels between 1% and 5%) report a lower number of symptomatic joint bleeding episodes than subjects with severe HA, and, as a result, they are expected to develop joint damage less frequently [2,9,10]. However, specific subgroups of people with mHA may show a more severe bleeding phenotype, particularly those with FVIII levels <3% [11,12]. Moreover, the contribution of subclinical bleeding events should be considered as causative of arthropathy [13]. The reported prevalence of arthropathy in people with mHA is widely variable (from 15%-77%), and prophylactic replacement treatment is prescribed in about 30% of cases, usually after the diagnosis of clinically overt arthropathy [14]. Nevertheless, treatment guidelines for mHA are scant and heterogeneous, and prophylaxis is often started late in mHA, thus leading to undertreatment and a not negligible prevalence of joint disease [1,[15], [16], [17]]. In the last few years, point-of-care ultrasound (PoCUS) has been more and more extensively adopted for the screening of hemophilic arthropathy [18]. The Hemophilia Early Detection by UltraSound (HEAD-US) system represents a standardized scanning protocol able to provide information about osteochondral damage (represented by chondral abnormalities and subchondral bone damage) and disease activity (expressed by the presence of synovial proliferation) in people with hemophilia [18]. Whereas the number of studies using PoCUS for joint assessment in people with severe hemophilia is growing [19], few data are available about mHA [15]. The aims of the present study were i) to evaluate joint health status in people with mHA receiving on-demand treatment; ii) to investigate the agreement between PoCUS performed according to the HEAD-US score and the clinical examination performed according to the Hemophilia Joint Health Score (HJHS).

## 2 Methods

### 2.1 Clinical and ultrasound examination

The study protocol for this multicenter, observational study has been approved by the Federico II University local Ethical Committee. The study was designed and conducted according to strengthening the reporting of observational studies in epidemiology checklist guidelines for an observational cross-sectional study [20].

Consecutive people with mHA (FVIII from 1% to <5%) were screened for inclusion by participating in hemophilia treatment centers. The exclusion criteria were i) the presence of active hepatitis B Virus, hepatitis C Virus, and HIV viral infection; ii) a current/previous history of anti-FVIII inhibitors; iii) known rheumatologic diseases; iv) concomitant treatment with anticoagulant or antiplatelet drugs; v) the presence of bleeding disorders other than mHA; and vi) presence of clinical signs of joint acute bleeding or recent episode of joint acute bleeding (in the last 4 weeks). In addition, we excluded patients with previous or current clinical indication for prophylactic replacement treatment and included only mHA receiving on-demand treatment based on treating physician’s clinical judgment. After the informed consent signature, major clinical and demographic characteristics of patients were collected (ie, age, FVIII levels, body mass index, annual bleeding rate (ABR) [21], and annual joint bleeding rate [AjBR]), and all enrolled patients underwent a clinical assessment of joint status for all the 6 major synovial joints (elbows, knees, and ankles) according to the HJHS protocol [22].

Clinical examination was performed by one investigator in each center (orthopedic surgeon, rheumatologist, or physiotherapist) who was an expert in the use of the HJHS tool. To be as conservative as possible, based on a study of healthy subjects, a 0 to 1 HJHS score was considered normal [23,24].

On the same day of the HJHS examination, all patients underwent a PoCUS assessment according to the HEAD-US protocol performed by a trained investigator blinded (hematologist or radiologist) to the HJHS results.

All PoCUS examinations were performed using a wideband high frequency (7-12 MHz) linear probe.

Each variable (synovial hypertrophy, chondral abnormalities, and subchondral bone damage) was scored according to the HEAD-US system, and the final score was calculated in an additive manner [18]. The presence of effusion, although easily detectable with PoCUS, was not scored because it is a transitory fluctuating finding and cannot express the status of a joint [25]. As an ancillary exploratory outcome, any change in the treatment schedule suggested by the treating physician after joint status assessment was recorded.

### 2.2 Statistical analysis

Continuous data were expressed as mean ± SD; categorical variables were expressed as percentages. To compare continuous variables, an independent sample *t*-test was used, and correlations were assessed using Pearson’s coefficients (*r*). The chi-squared test was employed to analyze categorical data. In case variables had a skewed distribution, results were expressed as median values with IQR, and comparisons were performed with the Mann–Whitney U-test. All the results were presented as 2-tailed values with statistical significance for *P* values <.05.

Agreement between the total HJHS and total HEAD-US score was evaluated by calculating the intraclass correlation coefficient (ICC). The agreement between the 2 systems in the detection of pathological joint changes was assessed by means of κ statistic. Agreement was judged poor for ICC or κ from 0 to 0.20; fair from 0.21 to 0.40; moderate from 0.41 to 0.60; good from 0.61 to 0.80; and very good from 0.81 to 1.00 [26].

Statistical analysis was performed using SPSS Statistics 29 (IBM SPSS Statistics).

## 3 Results

Among 60 people with mHA screened, a total of 51 subjects were included in our study, and 306 joints were evaluated by HEAD-US and HJHS examinations. Clinical and demographic characteristics of the study population are reported in the Table. The median ABR in the study population was 2.0 (IQR, 1.0-3.0; minimum-maximum [min-max], 0-15), with an AjBR of 1.0 (IQR, 0.0-3.0; min-max, 0-5).

**Table:** Demographic and clinical characteristics of the study population.

Clinical variable	Study population (*N* = 51), mean ± SD^a^
Age (y)	32.4 ± 18.8
FVIII levels (%)	2.68 ± 1.24
No. of bleedings/y, median (IQR)	2.0 (1.0-3.0)
No. of hemarhrosis/y, median (IQR)	1.0 (0-3.0)
BMI (kg/m^2^)	25.8 ± 3.98
HJHS, median (IQR)	2.0 (0.0-3.0)

BMI, body mass index; FVIII, factor VIII; HJHS, Hemophilia Joint Health Score.

a Unless otherwise indicated.

**Figure 1:**
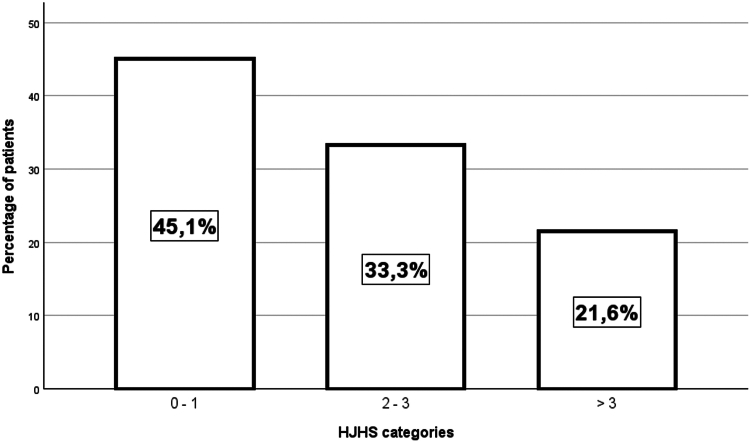
Distribution of Hemophilia Joint Health Score (HJHS) values in the study population.

The median HJHS was 2.0 (IQR, 1-3.0; min-max, 0-22). As shown in Figure 1, a 0 to 1 total HJHS score was found in 23 patients (45.1%), a 2 to 3 score in 17 (33.3%), and a >3 score in 11 (21.6%).

**Figure 2:**
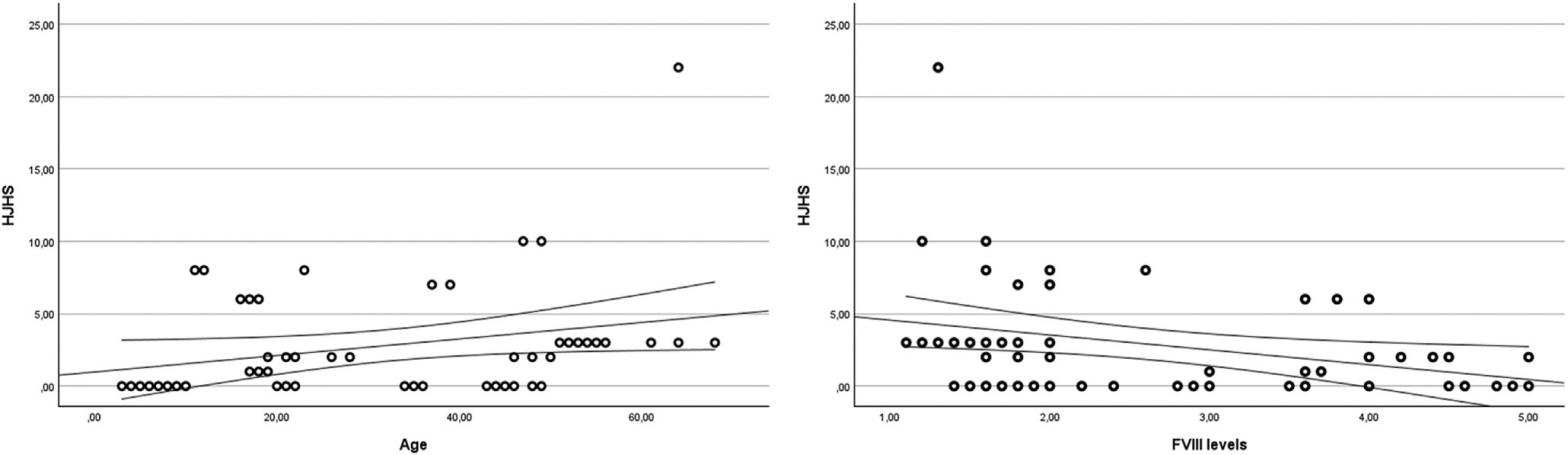
Scatter plot of Pearson correlations between Hemophilia Joint Health Score (HJHS) values and age (left panel) and factor (F)VIII levels (right panel).

Interestingly, as shown in Figure 2, the HJHS score directly correlated with age (rho = 0.372; *P* = .007) and inversely correlated with FVIII levels (rho = −0.376; *P* = .007). In contrast, no correlation was found between the HJHS score and body mass index (*P* = .24), ABR (*P* = .23), and AjBR (*P* = .76).

When the PoCUS assessment was performed, the median HEAD-US score was 2 (IQR, 1-7; min-max, 0-16). Osteochondral damage was found in 11 patients (21.6%) and hypertrophic synovium in 15 patients (29.4%). More in detail, severe (grade 2) hypertrophic synovium was found in 9 subjects (17.6%).

**Figure 3:**
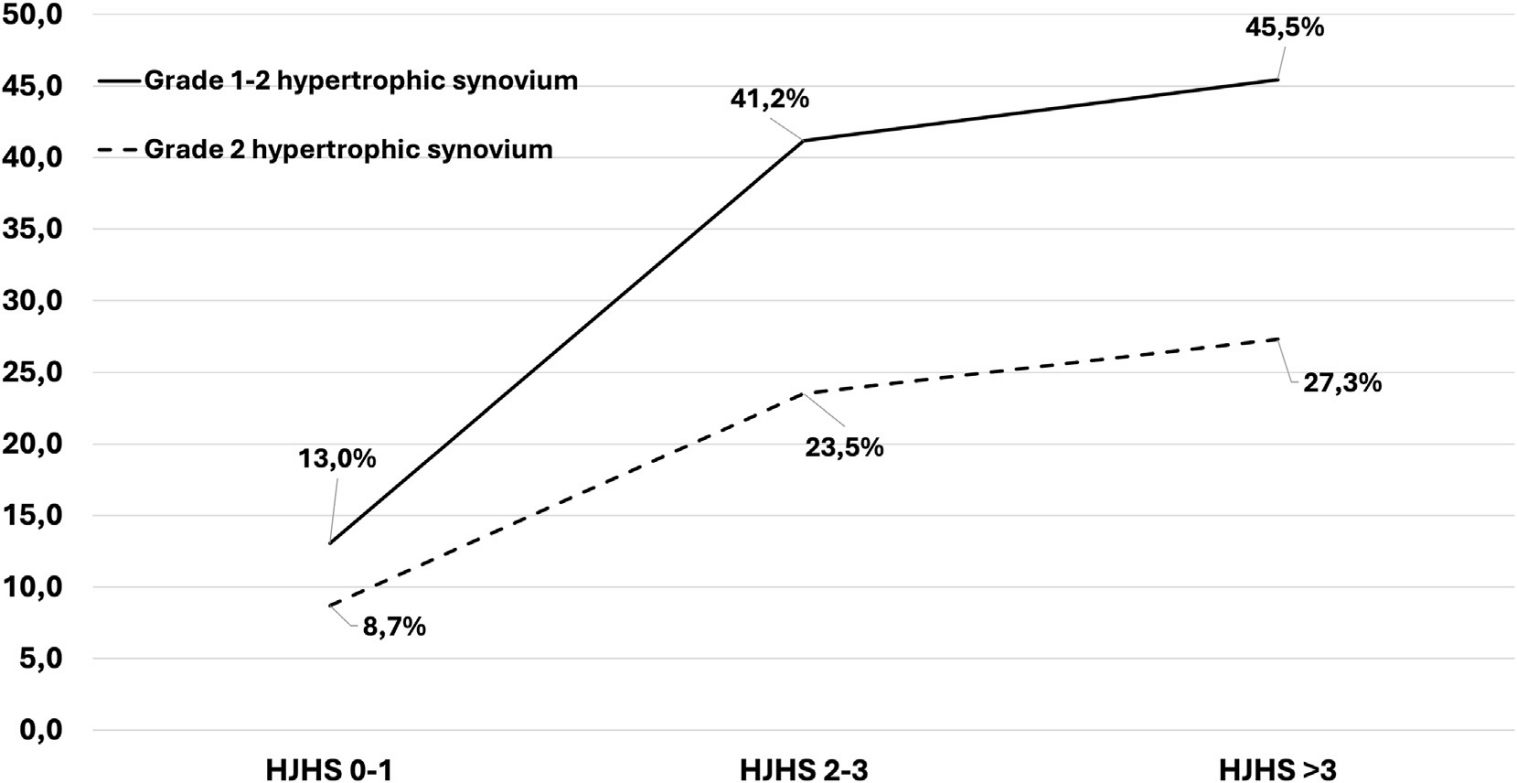
Prevalence of grade 1 to 2 hypertrophic synovium and grade 2 hypertrophic synovium on ultrasound in different Hemophilia Joint Health Score (HJHS) categories.

As shown in Figure 3, the prevalence of hypertrophic synovium in PoCUS progressively increased with an increasing HJHS score, with a statistically significant correlation between the 2 scores (rho = 0.732; *P* < .001). The ICC among the total HJHS and total HEAD-US was 0.811 (95% CI, 0.76-0.85), and the overall κ value for agreement between the 2 techniques in the detection of pathological joint changes was 0.652.

Interestingly, an inverse significant correlation was found between FVIII levels and the HEAD-US score (*r* = 0.345; *P* = .01).

Analyzing results by joint, hypertrophic synovium was detected in 8.8% of elbows, 6.9% of knees, and 11.8% of ankle joints, whereas degenerative alterations were detected in 6.9% of elbows, 10.8% of knees, and 12.7% of ankle joints.

After the joint health assessment, a change in the treatment schedule (from on-demand to prophylaxis) was suggested by the treating physicians for 19 people with mHA (37.3%). The most common reason for switching consisted of the detection of hypertrophic synovium in 11 out of 19 (57.9%) patients. A 3-time/week prophylaxis (35 IU/kg) with standard half-life FVIII concentrates regimen was suggested in 12 patients, a twice/week prophylaxis (35 IU/kg) in 2 cases, and twice/week prophylaxis (40 IU/kg) in 5 cases, followed by an accurate joint health status monitoring.

## 4 Discussion

Results of this cross-sectional study suggested that the prevalence of hemophilic arthropathy—including both osteochondral degenerative alterations and hypertrophic synovium—is not negligible in people with mHA receiving on-demand treatment. In addition, PoCUS was found to be able to detect signs of disease activity (ie, hypertrophic synovium) in clinically normal joints (HJHS 0-1). Arthropathy is the most common chronic complication in people with hemophilia, and, in recent years, growing attention has been paid to the early detection of disease activity, with the aim of preventing the development of clinically overt arthropathy [16,27]. Although people with mHA are expected to develop fewer joint damages than those with severe hemophilia [2,9,10], a widely variable prevalence of arthropathy has also been reported in this setting [14]. Currently, only few studies have been specifically designed to investigate joint health in mHA and were mostly limited to clinical examination [28,29]. Even though the HJHS is widely used for clinical assessment of joint health in people with hemophilia, its sensitivity and specificity in the identification of early stages of arthropathy and disease activity are widely questionable [19]. In light of this, HJHS alone may not be able to accurately evaluate the severity of joint deterioration [22,30]. Similarly, the Moderate Hemophilia (MoHem) study conducted on people with moderate hemophilia (A and B) showed that the HJHS is less accurate than the HEAD-US in detecting joint alterations [15], and early-stage joint abnormalities were detected by PoCUS in 5% of people with mHA with negative clinical examination [15].

In our study, we found a median HJHS of 2 (IQR, 0-3), with 45% of subjects with a score ranging from 0 to 1. Results of previous studies are widely heterogeneous, ranging from an HJHS of 0 (IQR, 0-2) in the PedNet cohort [31] to a score of 2 (IQR, 0-6) in the Dutch cohort [28], 4 (IQR, 1-10) in the MoHem study [15], and up to 7 (IQR, 4-11) in The Dynamic Interplay Between Bleeding Phenotype and Baseline Factor Level in Moderate and Mild Hemophilia A and B (DYNAMO) study [32]. Similarly, the ABR and AjBR values found in different studies were quite variable. These differences can be the expression of the extreme variability in the clinical phenotype of mHA. In addition, in our study, we specifically enrolled people with mHA receiving on-demand treatment according to treating physician’s indication, and this might have determined the exclusion of moderate patients with severe bleeding phenotype for which treating physician had previously suggested a prophylaxis regimen. On the other hand, another potential reason is the need for interobserver standardization for HJHS scoring [30].

When comparing the HJHS and HEAD-US, we found a significant direct correlation between the 2 scores, and we documented that the prevalence of PoCUS-detected hypertrophic synovium progressively increased with an increasing HJHS. Moreover, the assessment of the agreement confirmed a very good association between the 2 tools (ICC, 0.811), with a 65% κ value for the detection of pathological joint changes. However, when evaluating results analytically, it is interesting to highlight that PoCUS-detected osteochondral damage in ≈20% and hypertrophic synovium in ≈30% of the study sample and, more specifically, in ≈13% of patients with a negative HJHS. These data are in line with results from the paediatric network on haemophilia management cohort reporting hypertrophic synovium in 33% of cases despite a median HJHS of 0 and synovial changes in 21% of joints with a 0 to 1 HJHS [31].

Focusing on joint-level analysis, the ankles were the most frequently affected joints both by hypertrophic synovium and degenerative alterations. This could be due to the greater mechanical load than for the other joints, thus leading to an increased risk of subclinical and clinically overt bleeding episodes. Our PoCUS-based results are confirmed by magnetic resonance imaging data from the DYNAMO study [32] showing synovial hypertrophy in 15% of elbows, 3% of knees, and 53% of ankles, although the reported AjBR was 0, and 22% of patients never reported bleeding episodes [32]. Based on these results, more intensive monitoring of joint health and preventive treatment strategies should be warranted, particularly for people with moderate hemophilia [32].

From a clinical perspective, in addition to the bleeding phenotype, the detection of synovial hypertrophy, recognized as a marker of disease activity secondary to poorly controlled disease, suggests implementing and optimizing prophylaxis to prevent the progression of arthropathy [33]. Particularly, subclinical joint bleeds lead to synovial proliferation that could be often asymptomatic. Nevertheless, also in the absence of clinically overt bleeding, hemophilic arthropathy can occur [34]. Early detection of hypertrophic synovium before swelling becomes clinically evident may enable patients and caregivers to break the cycle by initiating prevention strategies to reduce continued joint damage, limit physical disability, and improve quality of life [35,36]. Similarly, the PoCUS examination identified signs of disease activity and degenerative alterations in some people with mHA and guided the treating physician’s decision-making process.

Although our study protocol did not include specific predefined criteria for treatment changes, the detection of pathologic PoCUS findings was accompanied by an indication to start prophylaxis in ≈37% of cases. This is particularly important in view of some evidence suggesting that prophylaxis may also be effective in preventing disease progression in people with moderate hemophilia [37], but it is used in only a minority of them [14].

Some limitations of the present study need to be discussed. First, only people with mHA receiving an on-demand replacement treatment have been included. This might have determined the exclusion of moderate patients with a severe bleeding phenotype for which treating physician may have previously suggested a prophylaxis regimen. However, this might somehow strengthen our results, suggesting that PoCUS has been able to identify signs of arthropathy in a perceived “low-risk” population. These findings confirm and extend the results of the MoHem and DYNAMO studies that included a more general population of people with mHA. Further interesting data might derive from the prospective follow-up of this study population, with the aim of evaluating the eventual progression of joint damage and verifying whether treatment change was associated with hypertrophic synovium resolution. Second, the reliability and repeatability of the results of the present study might be limited by the intra- and interobserver variability of the clinical examination (HJHS assessment) and PoCUS examination [33,38]. In particular, high variability in assessing HJHS is reported by several studies [39]; to limit this bias in our study, HJHS assessment was performed by well-trained personnel (>3 years of experience in the clinical examination of people with hemophilia). However, at variance with other ultrasound protocols requiring a long learning curve before obtaining an acceptable level of reproducibility, the HEAD-US score has been specifically designed to be easy to learn and use, thus allowing nonimaging expert physicians to perform PoCUS as a complementary part of the clinical examination [35]. Third, another potential limitation that needs to be discussed is the absence of a control group. Nevertheless, in the context of rare diseases such as hemophilia, few studies are designed with a control group. A comparison group is difficult to define, including both healthy volunteers and a cohort of patients affected by joint disease. First, according to a previous study evaluating joint status in a cohort of young people with hemophilia compared with young, healthy volunteers, the joints of young, healthy adults did not show hemophilia-specific abnormalities on magnetic resonance imaging [40]. Second, several data confirm that hemophilic arthropathy clinically and radiologically differs from osteoarthritis and rheumatic disease [2].

Overall, our results suggest that the presence of joint disease in people with mHA is not negligible and that PoCUS might represent a valuable tool for the identification of subjects who may take advantage of prophylactic treatment to prevent further disease progression. Integrating PoCUS into routine monitoring protocols for people with mHA could facilitate personalized care plans. Early detection of joint changes allows tailored interventions, potentially reducing or preventing the progression of hemophilic arthropathy. Longitudinal studies are warranted to assess the utility of ultrasound in tracking disease progression over time. Understanding how ultrasound findings correlate with clinical outcomes and long-term joint health will be crucial for establishing its role in comprehensive hemophilia care.

## 5 Conclusions

PoCUS is a promising, noninvasive, and readily available tool for the assessment of joint health in people with mHA. Early detection of joint changes may facilitate timely interventions, preventing the progression of hemophilic arthropathy. As we delve deeper into the era of personalized medicine, integrating PoCUS into the routine care of people with mHA could be a paradigm shift in optimizing their long-term outcomes.
